# Nutritional quality and marketing strategies of fast food children’s combo meals in Guatemala

**DOI:** 10.1186/s40608-016-0136-y

**Published:** 2016-12-08

**Authors:** Sofia Mazariegos, Violeta Chacón, Adam Cole, Joaquin Barnoya

**Affiliations:** 1 Cardiovascular Surgery Unit of Guatemala, 6a Ave 8-71 zona 10, Clinica #3, Ala Sur, Guatemala, 01011 Guatemala; 2 Center for the Prevention of Chronic Diseases, Instituto de Nutrición de Centro America y Panama, Guatemala, Guatemala; 3 School of Public Health and Health Systems, University of Waterloo, Waterloo, Canada; 4 Division of Public Health Sciences, Department of Surgery, Washington University in St. Louis, St. Louis, USA

**Keywords:** Fast food, Children, Obesity, Low/middle-income countries

## Abstract

**Background:**

Overweight and obesity prevalence in children is now on the rise in low/middle-income countries, including Guatemala. Fast food consumption is a recognized contributing factor to this rise. Fast food restaurants use health claims, toy giveaways, price incentives and fast service to promote children’s combo meals. This study sought to assess the use of toy giveaways, time to delivery and price incentives as marketing strategies in fast food chain restaurants in Guatemala. In addition, we sought to compare nutritional quality of combo meals with and without health claims.

**Methods:**

We visited one restaurant from each of the 8 major fast food chains in Guatemala and purchased all children’s combo meals to assess the prevalence of toy giveaways, health claims, and difference in delivery time and price between the combo meal and each meal item purchased separately. Each item was then classified as “healthy” or “less healthy” using the UK Nutrition Profile Model. Nutrition information was collected on-site, from the restaurant website, or by calling the customer service phone number.

**Results:**

We found 114 combo meals, 21 (18.4%) of which were children’s combo meals. Five (24%) had nutrition information, all were classified by our analysis as “less healthy”, and three had a health claim. On average, combo meals were US$1.93 less expensive than purchasing children’s meal items individually (*p* = 0.01). Time to delivery was 1.44 min faster for combo meals compared to purchasing meal items individually (*p* = 0.19).

**Conclusions:**

Children’s fast food combo meals in Guatemala were promoted using several marketing strategies that encourage consumption, including offering toy giveaways and price incentives. In addition, nutrition information is lacking in fast food chain restaurants. Public health advocates in Guatemala should consider a comprehensive approach to encourage healthier choices within fast food restaurants including policies that require fruit and vegetable options for meal side dishes, accessible and easy to read nutrition information, and restrict the use of toy giveaways.

**Electronic supplementary material:**

The online version of this article (doi:10.1186/s40608-016-0136-y) contains supplementary material, which is available to authorized users.

## Background

Globally, more than 41 million children under the age of five are overweight [1]. In 2009, the prevalence of overweight and obesity among Guatemalan school-age children was 27.1% and 7.5%, respectively (self-reported heights and weights) [2]. Excessive intake of energy-dense foods and reduced physical activity are major contributors of childhood obesity.

Children’s fast food consumption is associated with high energy, sodium, and saturated fat intake [3, 4], and may be a contributing factor to the growing obesity epidemic [5]. To promote consumption and influence food choice, fast food restaurants may use potentially misleading health claims [6] and offer toy giveaways [7, 8]. Caregivers perceive them as the marketing strategy that most influences the decision to purchase less healthy foods [7]. Additionally, fast food restaurants also use convenient combo meals, price incentives, and prompt delivery as marketing strategies [9]. However, most research on these marketing strategies is from high income countries, and is lacking from low/middle-income countries (LMICs) where the obesity epidemic is rapidly spreading.

Despite the overwhelming fast food marketing strategies targeting children, few jurisdictions have implemented marketing restrictions. Two cities in the United States (San Francisco and Seattle) have recently made efforts to restrict toy giveaways and implement menu labeling policies to help consumers make healthier choices [10–13]. Similar to California, New York City has proposed a policy that requires children’s combo meals with toys or promotional items to meet certain nutritional criteria [14]. Although preliminary evidence on the San Francisco toy ordinance does not show that fewer children receive toy giveaways with their combo meals, restaurants offer healthier default side dishes and drinks. This has led to a decrease in calories per order purchased by children [15].

In Guatemala, the availability of nutrition information and quality of children’s fast food combo meals has not yet been documented. Furthermore, there is no evidence of the prevalence of health claims on children’s combo meals, time to delivery and price incentives as marketing strategies. Therefore, we sought to assess the use of toy giveaways, time to delivery and price incentives on combo meals in fast food chains in Guatemala City. In addition, we sought to compare nutritional quality of children’s combo meals with and without health claims.

## Methods

All (8) major fast food restaurant chains located in Guatemala City, the largest and capital city of Guatemala, were surveyed. Two fast food restaurant chains did not offer children’s combo meals and were not included. Therefore the six fast food restaurant chains included were McDonald’s, Burger King, Wendy’s, Pollo Campero (local fried chicken), Kentucky Fried Chicken, and Pizza Hut.

We visited one restaurant (conveniently selected) from each chain between 12:00 PM and 3:00 PM over a 2 week-period. We counted the total number of lunch combo meals and those that were child-oriented (including toy giveaways). We considered children’s combo meals those that were marketed specifically to children. Children’s lunch combo meal packages could have the word “kids”, a picture of children, or a licensed character (e.g., Spiderman). Each children’s combo meal contained an entrée, side dish, beverage, and dessert. All children’s lunch combo meals that were listed on the menu board were purchased. We did not purchase any additional meal items or super-sized portions. The first brand and type of beverage included in the combo meal and offered by the cashier was purchased. We then assessed time to delivery and price between the combo meal and the meal items purchased individually. Hamburger, chicken drumstick, and ham and cheese pizza combo meals were used for the time to delivery and price comparisons.

Nutrition information was requested at the point of sale, from the restaurant manager, by checking the restaurant website, or by calling the customer service phone number. We then classified combo meals as “healthy” or “less healthy” using the UK Nutrient Profile Model (NPM) [16]. This model measures the nutritional quality of each food or drink, considering the inclusion of both positive (e.g., protein and fiber) and negative (e.g., sugars and sodium) nutrients [16]. Less healthy foods have a score of 4 or higher and beverages 1 or higher [16].

We assessed if combo meals that included toys met international nutritional quality criteria. Two U.S. standards are available to determine if a fast food combo meal can include a toy giveaway, the Institute of Medicine (IOM) standard for the National School Lunch and Breakfast Program [17] and the Model Ordinance for Toy Giveaways at Restaurants developed by the National Policy & Legal Analysis Network (NPLAN) to Prevent Childhood Obesity [18]. Both are based on energy (calories), sodium (mg), trans fat (g) content, and the percentage of total energy from total and saturated fat. Neither standard includes total sugar; however, we compared the total sugar of combo meals in Guatemala with those that have been found in children’s combo meals in the U.S [19].

Health claims found on any package (i.e., paper wrapper or cups, children’s combo meal boxes) of the combo meal items were also counted. We defined a health claim as any text or figure stating that a food has a particular nutritional property including, but not limited to energy, protein, fat, carbohydrates, and vitamins or minerals (e.g., “A perfect day to taste the flavour of vitamins in fruits”) [20]. We then compared the nutrient content of combo meals with and without claims.

REDCap™ web-based application was used for data entry and STATA (version 13.0, 2013) for statistical analysis. Median price (interquartile range, IQR), delivery time, and NPM (for those with nutrition information) were calculated.

## Results

We found six different fast food restaurant chains that offered children’s combo meals with toy giveaways. The fast food restaurant chain that had the most restaurants was Pollo Campero (Table 1).

**Table 1:** Fast food chain restaurants in Guatemala^1^

	n	Guatemala City, n (%)
McDonald’s	77	41 (53.2)
Burger King	42	35 (83.3)
Wendy’s	9	8 (88.9)
Pollo Campero	126	75 (59.5)
KFC	3	3 (100.0)
Pizza Hut	39	37 (94.9)

^1^Data from restaurant’s websites, accessed on November, 2013

**Fig. 1:**
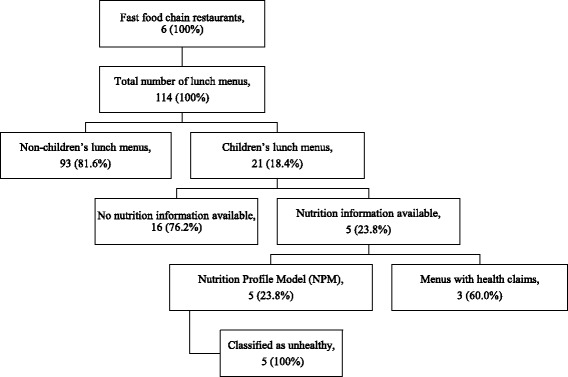
Children’s fast food lunch combo meals in Guatemala

A total of 114 combo meals across all 6 chains were identified and children’s combo meals per restaurant ranged (Fig. 1) from 9.5% to 25% (*p* = 0.85) of all combo meals available (Table 2). Median price was US$3.60 (IQR 3.31 to 3.90, *p* = 0.15). On average, combo meals were less expensive (US$1.93, *p* = 0.01) and took less time (1.44 min, *p* = 0.19) to be delivered compared to purchasing meal items separately. All restaurants offered a soft drink as the first drink option and all combo meals included a toy giveaway.

**Table 2:** Delivery time, price, and nutrition information availability of children’s combo meals in fast food chain restaurants. Guatemala City, Guatemala

		Children’s combo meals^1^							
Restaurant	Total combo meals, (n)							Combo meals with nutrition information (n)%	
		Children’s combo meals, n (%)		Delivery time					Source of information
			Price (US$)	(Minutes) ^3,4^		Price (US$) ^5^			
			(Median, range) ^2^	Combo meal^6^	Individual items^6^	Combo meal^6^	Individual items^6^		
McDonald’s	20	5 (25.0)	3.63 (3.51 – 3.64)	2.46	2.75	3.63	3.89	3 (60)	Restaurant Manager
Burger King	18	4 (22.2)	3.53 (3.31 – 3.79)	2.03	2.63	3.53	4.41	2 (50)	Restaurant Manager
Wendy’s	23	4 (17.4)	3.64 (3.64 – 3.64)	1.33	2.78	3.63	6.88	0 (0)	None
Pollo Campero	14	3 (21.4)	3.50 (3.50 – 3.50)	2.86	4.33	3.5	5.22	0 (0)	None
KFC	18	3 (16.7)	3.64 (3.64 – 3.64)	2.48	1.8	3.63	6.62	0 (0)	None
Pizza Hut	21	2 (9.5)	3.77 (3.64 – 3.90)	21.81	29.08	3.63	6.1	0 (0)	None

^1^Children’s combo meals were defined as those that were promoted for children and contained an entrée, side dish, beverage, and included dessert or toy giveaway

^2^ U.S. Dollars at an exchange rate of 7.70 Quetzales per 1 US$

^3^Time between placing the order and delivery

^4^p = 0.19

^5^p = 0.01

^6^ McDonald’s, Burger King, Wendy’s; Kentucky Fried Chicken, Pollo Campero; and Pizza Hut

We found that nutrition information was not easily accessible and only available for two out of six fast food restaurants. Five out of 21 children’s combo meals (Table 2) had nutrition information and all were classified as “less healthy” according to the NPM. Regarding the National School Lunch and Breakfast Program and the Model Ordinance for Toy Giveaways at Restaurants (Table 3), combo meals had more sodium, calories from fat, and saturated fat than either of the nutrition standards (not statistically significant). Moreover our results were similar to those of a study from the United States with a similar design (Table 3). Three of the children’s lunch combo meals included a health claim (Table 4, no statistically significant difference).

**Table 3:** Nutrition information for selected Guatemalan children’s combo meals with nutrition standards and previous research. Guatemala City, Guatemala (*n* = 5)

Nutrient	Guatemalan children’s combo meals, Median (Range)	Institute of Medicine Nutrition Standard^1^	National Policy & Legal Analysis Network to Prevent Childhood Obesity Nutrition Standard^2^	U.S. children’s combo meals, Median (Range)^3^
Energy (kcal)	514 (404 – 725)	<650	<550	530 (180 – 880)
Sodium (mg)	885 (495 – 1173)	≤640	<640	810 (340 – 1960)
Total Sugar (g)	46 (36 – 52)	-	-	37 (0 – 78)
Saturated fat (%)	11 (8 – 13)	<10	<10	9 (0 – 18)
Calories from fat (%)	39 (23 – 52)	<35	<35	34
Trans fat (g)	0.0 (0 – 0)	<0.0	<0.5	-

^1^IOM Nutrition Standards for the National School Lunch and Breakfast Program for children 5 to 11 years of age

^2^Model Ordinance for Toy Giveaways at Restaurants

^3^from O’donnell et al. Am Am J Clin Nutr 2008 Nov; 88(5): 1388-95Developed by the National Policy & Legal Analysis Network to Prevent Childhood Obesity (NPLAN)

**Table 4:** Nutrition information for children’s combo meals with or without a health claim. Guatemala City, Guatemala

	Health claim	
	Yes	No
Nutrient	(*n* = 3)	(*n* = 2)
Energy (kcal)^1^	464 (404 – 514)	705 (685 – 725)
Sodium (mg)	655 (495 – 885)	1058 (943 – 1173)
Total Sugar (g)	52 (46 – 52)	36 (36 – 36)
Saturated Fat (%)	11 (8 – 13)	9 (8 – 11)
Calories from fat (%)	41 (39 – 52)	25 (23 – 26)
Trans fat (g)	0.0 (0 – 0)	0 (0 – 0)

^1^Median (range) unless otherwise noted

## Discussion

According to our findings, nutrition information was not available for most combo meals offered targeting children. The few that did have information, the nutrition quality was not optimal according to U.S. nutrition standards [19]. Furthermore, fast food chains are using toy giveaways that may promote less healthy combo meals to children. Comprehensive approaches are needed to improve access to healthy options within fast food restaurants and make these more desirable to children.

Marketing strategies found in our study (i.e., toy giveaways, time to delivery, price incentives, and health claims) are widely used by fast food chains [8, 21]. Basch et al. [22] found that these promoted reduced cost combo meals with high sugar, sodium, and fat content. Given that fast food combo meals with a poor nutritional content contribute to the increase of childhood obesity [3], restrictions are needed to ensure that nutrient quality of children’s fast food combo meals meet healthy guidelines. For instance, Guatemala could implement a policy that requires additional fruit and vegetable options for combo meal side dishes and low-fat milk as the first beverage alternative. To increase uptake, these could be offered as the default option rather than french fries and a soft drink. This would likely improve combo meal nutritional quality.

Fast food restaurants offer combo meals as an efficient and convenient way to purchase a meal. According to our findings, chains in Guatemala (and most likely elsewhere) offer meal items that are less expensive and served faster when they are purchased in a combo meal rather than separately. This suggests restaurants are using combo meals to offer more food for lower prices, promoting consumption and therefore higher energy intake.

Menu labeling is now being explored as a strategy to reduce calorie consumption. In 2008, the Board of Health of the New York City Department of Health and Mental Hygiene implemented regulations mandating chain restaurants to include calorie information on menus [23]. Other U.S. cities and states have since tried to implement similar policies [24, 25], and the Patient Protection and Affordable Care Act (ACA) requires menu labeling at all restaurant chains with 20 or more locations nationally [26, 27]. Guatemalan fast food chains did not include calorie information on their menus and we found personnel were evasive when asked for nutrition information. Therefore, we were unable to obtain data for most of the combo meals we found. Mandatory menu labeling is a promising strategy providing consumers with knowledge [28] and improving the nutrient content of fast food combo meals since it could encourage restaurants to reformulate their products to offer healthier options. Rationale in favor of menu labeling is grounded in considerable evidence and unintended consequences are unlikely [29]. The Guatemalan Ministry of Health should support policies requiring fast food chains to provide nutrition information at the point of sale and on menus in order to support the selection of healthier options. Furthermore, this information needs to be presented in a way that is easy to understand for consumers regardless of literacy level.

Consumption of nutrient poor foods, such as fast food combo meals, promoted by toy giveaways is likely one of the contributing factors to the observed increase in childhood obesity [30]. Children’s combo meals found in our study included a toy and those that had nutrition information failed to meet nutrition standards proposed by the IOM and in California. Guatemala lacks regulation to improve the nutritional quality of children’s combo meals. However, restricting toy giveaways to children’s combo meals that meet established nutrition standards is likely to encourage healthier combo meal selections [8, 31].

Health claims create a “halo” effect over food, preventing consumers from seeking further nutrition information [32]. Likewise, consumers also draw inferences about the nutritional quality of food with health claims on the package or combo meal [33]. The food industry, however, is not the only industry using claims as a marketing strategy. The tobacco industry uses terms like “light” and “smooth” to give the impression that cigarettes are less harmful [34]. Our results yield that most combo meals included in our study had health claims, even though they were all classified by our analysis as ‘less healthy’. Therefore, nutrient content or nutritional quality should be required in order to include health claims in children’s fast food combo meals to guarantee accuracy and avoid misleading marketing.

Our study has strengths and limitations. To the best of our knowledge this is the first study to document the prevalence, marketing strategies, and nutritional quality of children fast food combo meals in a LMIC. In addition, we surveyed local and international fast food chains. However, we only included children combo meals and therefore our findings are not generalizable to all combo meals available at fast food chains. In addition, we did not evaluate how these strategies influence purchasing decisions in Guatemala.

## Conclusions

In conclusion, given our findings, Guatemalan public health authorities (and elsewhere) should consider a comprehensive approach to encouraging healthier choices within fast food restaurants. Policies are required to include fruit and vegetable options for meal side dishes and healthier beverage alternatives. Furthermore, policies are required to mandate fast food chains to provide easily accessible and understandable nutrition information for combo meals and restrict the use of toy giveaways. Once implemented, research is warranted to evaluate the implementation and impact of these policies on childhood obesity rates in Guatemala.

## Additional files

Additional file 1: Child-oriented fast food meals in Guatemala. Data on prevalence of child-oriented fast food meals in Guatemala (XLSX 36 kb)
Additional file 2: Price and nutrition information of child-oriented fast food meals in Guatemala. Data on price and nutrition information of child-oriented fast food meals in Guatemala. (XLSX 53 kb)

## Abbreviations

LMIC: Low/middle-income country
NPM: Nutrient profile model
